# Clinical and histopathological responses to bee venom phonophoresis in treating venous and diabetic ulcers: a single-blind randomized controlled trial

**DOI:** 10.3389/fmed.2023.1085544

**Published:** 2023-04-20

**Authors:** Eman M. Othman, Hamada Ahmed Hamada, Ghada I. Mohamed, Ghada A. Abdallah, Zeinab S. Ahmed, Abdullah M. Al-Shenqiti, Ahmed Mahmoud Kadry

**Affiliations:** ^1^Department for Surgery, Faculty of Physical Therapy, Cairo University, Cairo, Egypt; ^2^Department for Biomechanics, Faculty of Physical Therapy, Cairo University, Cairo, Egypt; ^3^Department of Basic Science, Faculty of Physical Therapy, Cairo University, Cairo, Egypt; ^4^Department of Cardiovascular, Respiratory Disorder and Geriatrics, Faculty of Physical Therapy, Cairo University, Cairo, Egypt; ^5^Faculty of Medical Rehabilitation Sciences, Taibah University, Medina, Saudi Arabia; ^6^Faculty of Physical Therapy, Kafrelsheikh University, Kafr el-Sheikh, Egypt

**Keywords:** diabetic foot ulcer, venous ulcer, chronic leg ulcer, phonophoresis, bee venom

## Abstract

**Introduction:**

Chronic venous and diabetic ulcers are hard to treat that cause patients long time of suffering as well as significant healthcare and financial costs.

**Purpose:**

The conducted study was to evaluate the efficacy of bee venom (BV) phonophoresis on the healing of chronic unhealed venous and/or diabetic foot ulcers Also, to compare the healing rate of diabetic and venous ulcers.

**Methodology:**

The study included 100 patients (71 males and 29 females) with an age range of 40-60 years' old who had chronic unhealed venous leg ulcers of grade I, grade II, or diabetic foot ulcers with type II diabetes mellitus. They randomly assigned into four equal groups of 25: Group A (diabetic foot ulcer study group) and group C (venous ulcer study group) who both received conservative treatment of medical ulcer care and phonophoresis with BV gel, in addition to group B (diabetic foot ulcer control group) and group D (venous ulcer control group) who both received conservative treatment of medical ulcer care and received ultrasound sessions only without BV gel. Wound surface area (WSA) and ulcer volume measurement (UVM) were used to assess the ulcer healing pre-application (P_0_), post-6 weeks of treatment (P_1_), and after 12 weeks of treatment (P_2_). In addition to Ki-67 immunohistochemistry was used to evaluate the cell proliferative in the granulation tissue of ulcers pre-application (P_0_) and after 12 weeks of treatment (P_2_) for all groups.

**Results:**

This research revealed a statistical significance improvement (p ≤ 0.0) in the WSA, and UVM with no significant difference between study groups after treatment. Regarding Ki-67 immunohistochemistry showed higher post treatment values in the venous ulcer group in comparison to the diabetic foot ulcer group.

**Conclusion:**

Bee venom (BV) provided by phonophoresis is effective adjuvant treatment in accelerating venous and diabetic foot ulcer healing with higher proliferative effect on venous ulcer.

**Clinical trial registration:**

www.ClinicalTrials.gov, identifier: NCT05285930.

## Introduction

Acute and chronic wounds can be distinguished. Acute wounds are caused by burns, surgery, or trauma, but chronic wounds, such as arterial, diabetic, pressure, and venous ulcers, are caused by an underlying pathophysiological condition, such as vascular insufficiency (1). Chronic diseases, such as vascular disease and diabetes, are becoming more common worldwide (2). There has also been an increase in the number of patients who are at risk of developing chronic severe wounds. Chronic wounds are non-healing due to a variety of factors, such as pressure, venous, arterial, and diabetic neuropathies, and healing of these types of wounds does not happen in a quick, efficient, and orderly manner. Chronic wounds frequently remain static in the inflammatory phase, providing a sclerotic, sloughy, and impeded environment for bacterial proliferation. These wounds can rapidly become infected and are hard to treat. Before a wound may progress to healing and closure, effective wound debridement and disinfection procedures are required (3). Adults with chronic ulceration of the lower legs experience growing discomfort, friable granulation tissue, foul odor, and wound collapse rather than healing. As a result, there is community suffering as well as significant healthcare and financial costs (4). According to the Wound Healing Society, venous stasis ulcers, diabetic (neuropathic) foot ulcers, and pressure ulcers (bedsores) affect around 15% of elderly Americans. It has been claimed that of all leg ulcer presentations, venous ulcers account for 70% of cases, arterial disease accounts for 10%, and ulcers of mixed etiology account for 15% of cases (5). The other 5% of leg ulcers are caused by less common causes, including trauma, diabetes, pressure ulcers, atherosclerosis, tuberculosis, and leprosy, all of which pose significant diagnostic, evaluation, and therapeutic challenges (6). Debridement of the necrotic tissue, use of moist dressings, and off-loading are all primary wound care procedures (7). However, these passive treatments have had varying degrees of success (8), with amputation values as high as 29%, especially in diabetic foot ulcers after 5 years (9). As a result, there is a clear need for more advanced wound healing therapies as well as novel diagnostics to enhance healthcare decisions (10).

In the animal realm, bee venom is a unique weapon. The venom machinery of bees plays a critical function in the colony's defense. Bee venom contains a highly effective and complicated mix of chemicals that protects bees from a wide range of predators, including other arthropods and vertebrates (11). Several peptides have been found in bee venom from the venom gland in the abdomen cavity (12), including melittin, apamin, adolapin, the mast cell-degranulating peptide, enzymes (phospholipase A2), physiologically active amines (i.e., histamine and epinephrine), and non-peptide components with medicinal capabilities (13). Because of its extensive range of pharmacological properties, bee venom is commonly employed in Eastern medicine (14). Furthermore, it has been used in a variety of products as a cosmetic ingredient with anti-aging, anti-inflammatory, and antibacterial characteristics (15). According to researchers, BV has therapeutic benefits for many skin problems, such as eczema, dermatitis, psoriasis, furunculosis (recurring boils), cicatrices, baldness, acne, and others (16). Bee venom's biological properties have been used to treat wounds. One study, which was carried out on mice, revealed that wound size was reduced while epithelial proliferation increased. In animal models, the topical application of bee venom is effective, particularly in reducing the size of wounds (17).

Depending on the disease, BV therapy can be administered *via* cream, liniment, or ointment application, injection, acupuncture, or even directly through a live bee sting (18). The usage of bee venom (BV) on an acupoint generated a significantly more potent anti-inflammatory and antinociceptive effect than the usage on a non-acupoint (16). Traditional BV therapy consisted of direct bee stings, which caused pain and inflammation as well as no control over the actual dose, resulting in poor patient satisfaction, or BV injections into acupoints, which is an invasive technique that causes extreme pain (19). For these reasons, the need for another method of applying BV is critical.

Phonophoresis is the use of ultrasound to increase skin absorption and infiltration of topical medications into deep tissues. Factors such as percentage, quantity, drug penetration depth of the skin, and potential drug toxicological risks to the tissues all influence how therapeutic drugs are administered. In phonophoresis, local anesthetics, anti-irritants, and anti-inflammatory drugs are used. Phonophoresis is a non-invasive, painless treatment that has minimal side effects, is well-tolerated, and has been used to treat musculoskeletal and dermatologic disorders (20). Phonophoresis is a non-invasive technique that uses ultrasound waves to improve transdermal drug delivery (21). TDD has several benefits over systemic approaches as it can be administered orally and intravenously (22). While the transdermal patch is a beneficial early TDD strategy, only small-molecule drugs can be absorbed due to the barrier action of the skin's stratum corneum (23). The gel is a jelly-like solid substance made up of extract and matrix. Gels have strong biocompatibility, can prolong the action, are linked to improved treatment outcomes, and are appropriate for polypeptide medicines (24).

Unfortunately, we observed contradictions of opinions about the best type of physical therapy approach for the healing of chronic ulcers. In the current study, we attempt to determine the effect of bee venom phonophoresis on accelerating the healing of chronic venous and diabetic foot ulcers. Second, we aim to compare the responses of different types of ulcers to the treatment.

## Materials and methods

### Participants

A total of 100 patients (men and women) with type II diabetes mellitus who had grade I and grade II (according to the Meggitt-Wagner classification) chronic unhealed venous leg ulcers located on their soles or leg, or grade I and grade II diabetic foot ulcers, and whose leg ulcer duration ranged from 3 to 6 months participated in this study. They were recruited randomly from the Department of Surgery of the Al Kasr El Aini Hospital in Cairo and/or from the Outpatient Surgery Clinic in the Faculty of Physical Therapy-Cairo University. The diagnosis before their enrollment into the study was made clinically by physicians. Eligible patients (71 men and 29 women) were in the age group of 40–60 years. They had not undertaken any other physical therapy modality for healing ulcers, and all of the participants were non-smokers, taking prescribed medications, and following controlled diet therapy as prescribed by their physicians. All patients in the diabetic foot ulcer groups (A and B) were non-insulin-dependent and controlled blood glucose levels. Patients with life-threatening conditions, such as renal failure and myocardial infarction and/or any systemic diseases that could interfere with the study's objectives, were excluded, as were patients with skin disease and/or any disease that can lead to ulcers rather than diabetes, such as varicose veins, trauma, peripheral vascular diseases, and/or active malignancy. Those with ulcers with a surface area of <2 cm^2^ or more than 8 cm^2^ were excluded from the study.

### Research design

The study design was a parallel, pre-/post-test, prospective, randomized, single-blind, and controlled experiment. Before the start of the study, ethical approval was acquired from the Institutional Review Board with the number P.T.REC/012/003573, and the study was registered in the Clinical Research Registry under the ClinicalTrials.gov ID: NCT05285930. We followed the Declaration of Helsinki Guidelines for Human Research while conducting this study. The consent form, intervention, and methods of assessment were also accepted by Cairo University's Faculty of Physical Therapy's Ethical Committee Board. The contributors were aware of the goal, research advantages, their right to resign at any moment, and the suppression of information. The study started in January 2021 and ended in April 2022.

### Randomization

**Figure 1:**
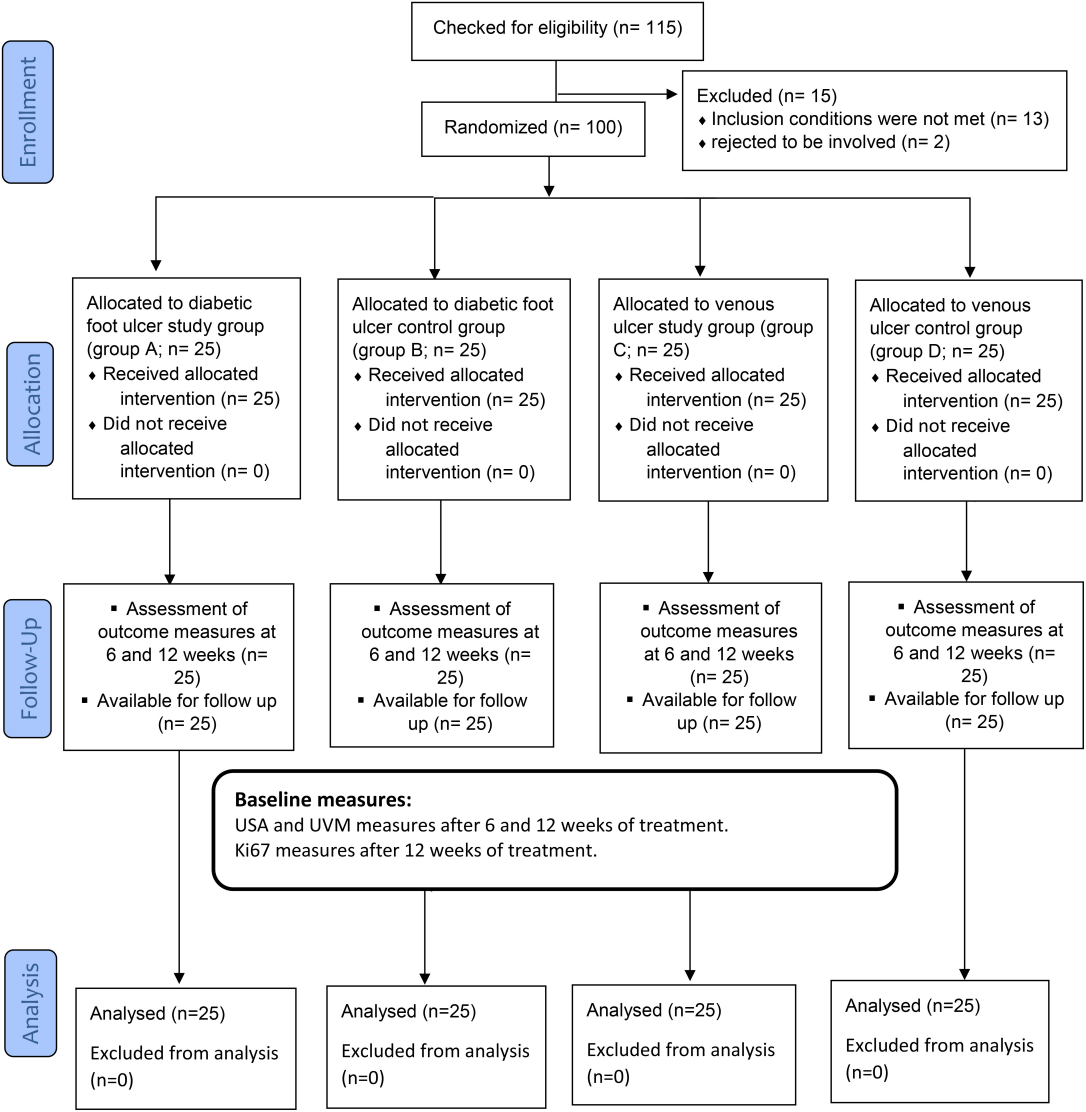
The flowchart of the trial.

Each participant provided informed consent after being given clear instructions about the nature, goals, and advantages of the research, as well as their ability to decline or withdraw at any stage and the confidentiality of any gathered information. Through the coding of every piece of information, anonymity was ensured. To minimize bias and variance between the three groups, patients were randomly allocated with a 1:1 distribution ratio. An independent author who was blinded to the randomization process randomly allocated them to one of the four groups (A, B, C, or D) by opening sealed envelopes containing a computer-generated randomization card. To guarantee hidden distribution, randomization codes were kept secret in sealed dark envelopes and were consecutively numbered. After randomization, no volunteers withdrew from the trial, as shown in Figure 1. Group A (diabetic foot ulcer study group) group C (venous foot ulcer study group) and each group comprised 25 patients who received conservative treatment of medical ulcer care and 25 of them who received phonophoresis with BV gel, while group B (diabetic ulcer control group) group D (venous foot ulcer control group) included 25 patients who received conservative medical ulcer care and ultrasound sessions with essential gel but no BV gel. On the occasion that a participant had both ulcerated legs in the trial, each leg was randomized independently.

### Interventions

The first author initiated the intervention according to the patient-assigned group after completing the pre-intervention evaluations. The treating authors and two of the staff physical therapists from the faculty of surgery unit's outpatient clinics performed the intervention. All physical therapists who collaborated with patients were required to be well-versed in bee venom phonophoresis techniques. To standardize the protocol and therapy, they also attended a 1-h training session led by the primary author.

### Outcome measures

At the outset of the trial, participants' personal information was gathered, including age, sex, location of ulcers, the elapsed time from ulcer injury, and comorbidity-associated conditions. The third author, who was blinded to randomization, examined all the quantifiable clinical outcomes before, after 6 weeks, and after the 3rd month of the intervention program. Wound surface area (WSA) and ulcer volume measurement (UVM) were the clinical outcome measures used to assess the ulcer healing process pre-application (P_0_), after 6 weeks of treatment (P_1_), and after 12 weeks of treatment (P_2_) for the four groups included in the study. In addition, the pathological ulcer granulation tissue proliferation rate was assessed using Ki-67 immunohistochemistry pre-application (P_0_) and after 12 weeks of treatment (P_2_) for the four groups.

### Wound surface area (WSA) in cm^2^

The wound surface area (WSA) was estimated by covering the ulcer with a piece of cleaned transparent film and using a fine-tipped transparency marker to trace the ulcer perimeter on the film. Each ulcer was measured with its own transparent film. The trace was then placed on the metric graph paper and the count of 1-mm squares within the perimeter was calculated (only the entire 1-mm squares were counted, and the area was converted to square centimeters) (19).

### Ulcer volume measurement (UVM) in cm^3^ (width x length x depth)

So that measurements could be taken, the patient was placed with the ulcer facing upward. To have the longest length and width, the ulcer was traced on translucent paper, which was then placed over the metric graph paper. A disposable measuring tape was directed into the deepest point of the ulcer to record the ulcer depth. The volume of the ulcer was calculated (width x length x depth) (20).

### Ki-67 immunohistochemical staining procedure and positive-rate scoring

#### Histology

While the participant was lying in a relaxed position, a wound tissue biopsy was obtained at the laboratory center for each patient in the four groups (A, B, C, and D) before treatment and after 12 weeks of treatment by taking a 1-mm skin biopsy from the boundary of the ulcer. The biopsy was then coded and placed in 10% buffered formalin, then handled in progressive grades of alcohol (70, 90, and 100% absolute) and xylene before being embedded in paraffin blocks, which were serially partitioned and stained with hematoxylin and eosin (H and E) stains (25). Immunohistochemical staining was carried out according to the standard procedure with a BenchMark GX autostainer (Ventana Medical Systems, Tucson, AZ, USA) using monoclonal antibodies against Ki-67 (MIB-1, Dako, Glostrup, Denmark) (26).

Deparaffinized samples were placed on a plastic rack in a basin having 250 ml of 10-mm citric acid (pH set at 6.0 utilizing 2 M sodium chloride), microwaved at 750 W (4-min pulses), and then left at room temperature for 10 min. The samples were then washed with Tris-buffered saline (TBS), drained, and the portion ringed with a risen pen before being cleaned in running tap water. A monoclonal mouse anti-human Ki-67 antigen was used to stain the slides (dilution 1:75–1:150). The slides were then rinsed three times in TBS, immersed in a biotinylated anti-mouse antibody (diluted 1:4000) for 1 h, cleaned three times in TBS, and AB Complex/Strept Ab Complex (K0355; Dako UK Ltd.) was administered for 1 h more. After 5 min, sections were washed in distilled water, rinsed under running tap water, and counterstained with hematoxylin for 60 s (Vector DAB kit, SK-4100; Vector Laboratories, Burlington, CA, USA). Then, they were cleaned one more time, dehydrated, and fixed with distyrene plasticizer xylene (DPX). The overall number of cell interfollicular basal and first suprabasal layer cells was counted using a high-powered light microscope (Olympus CX41) (25). The main antibody was left out of the negative control, and no fluorescence was recorded. The numbers of Ki-67 brightly stained cells and stained nuclei were manually recorded on 10 random areas for each test condition, and their results were reported in percentage as a ratio of Ki-67-stained cells to the overall cell number. The Ki-67 nuclear staining was seen as a favorable finding. Ariol-200 was used to count the positive nuclei (Leica Biosystems, Nussloch, Germany). Positive Ki-67 immunoreactivity showed up as nuclear brown staining. The Ki-67 index assessor was masked from patient information, including laboratory findings and quantitative biopsy findings (26).

### Treatment procedures

All patients in the four groups, A, B, C, and D, received the same conservative treatment for the ulcer, the same nursing care, the same medications, and prescribed diet. Compression bandaging, non-adherent dressings, debridement, and systemic antibiotics based on culture tests were the standard treatments for ulcers. Participants with exposed ulcers were treated three times a week with non-adherent dressing and compression bandaging until ulcer qualified nurse and surgeons used surgical tools, such as a scalpel, scissors, and curette, to perform, twice a week, sharp debridement as needed to remove dead necrotic tissues and foreign bodies. They were also responsible for performing the wound punch biopsy for the histopathological examination. Patients with venous ulcers who had cured ulcers were given type 2 elastic stockings to wear throughout the day. All participants received the same written and verbal instructions to elevate the injured limb and to exercise (21). The wound was irrigated with regular saline and then cleaned with Betadine solution one time a day. Dressings: After irrigation, the ulcer was covered with sterile Vaseline gauze (Sofra-Tulle dressing) and changed one time daily (22). In addition, for patients with diabetic foot ulcers, hypoglycemic medications other than insulin injections to control blood glucose levels were given.

Each patient was lying supine to undergo phonophoresis on the ulcer margins. Any clothes covering their legs or feet were removed. The ultrasound unit's connector was connected to the basic power supply. The efficiency of the ultrasonic applicator was checked regularly before and after each use on the patients. The check comprised all key acoustic output parameters (pressure, amplitude, and frequency) and sector pattern homogeneity (23). This sort of test of the applicators' acoustic production was necessary to remove the risk of device malfunction.

### Bee venom gel preparation

Bee venom solution (100 mg/mL) that was prepared and preserved at the correct temperature was purchased from the Egyptian Organization for Biological Products & Vaccines (Vacsera). The solution was a mix of a crude form of BV dissolved in sterile normal saline with a concentration ratio of 1:1 vol/vol. Then, the BV gel was prepared at the laboratories of the Faculty of Pharmacy, Badr University, Cairo, Egypt, by dissolving the previous mixture in 10% propylene glycol, followed by the addition of 0.01% butylparaben. To make bee venom gel, the resulting mixture was mixed with the matrix. The BV gel had a homogeneous and translucent appearance and a pH of 7.53. There was no discoloration, phase separation, or off-putting odor. Stratification was not observed after 30 min of centrifugation at 2,500 rpm at 25°C (17).

All participants were examined for BV allergy. A single clinical dose of diluted BV in normal saline, 0.05 ml (1 g/ml), was injected by either an intradermal or a subcutaneous route into the forearm. Individuals participated in this research if the examined injury produced a skin reaction that was round and smaller than 10 mm and erythema smaller than 26.5 mm for 10–15 min. Patients were given a previously prepared BV gel as a topical treatment (27, 28) three times per week using non-contact low-frequency pulsed ultrasound (Chattanooga Intelect MOBILE Model-2776, manufactured in Mouguerre, France) with a frequency of 1 MHZ, delivered at a spacing of 5–15 mm from the ulcer bed with a slight mist of sterile saline, with each session lasting 10 min (29). Each participant received a total amount of ~0.6 mg up to a maximum of 1 mg of BV gel at each session for a total of 10 min (30). For groups C and D, the participants received ultrasound using the same techniques as for groups A and B with plain gel instead of BV gel (31). Treatment was conducted during three sessions a week for 12 consecutive weeks.

### Statistical analysis and sample size

All statistical analyses were performed using IBM SPSS Statistics for Windows, version 25.0 (released in 2017), IBM Corp., Armonk, NY, USA. Descriptive statistics were employed to compare subject characteristics between groups. For categorical variables, the chi-squared test was utilized. Testing of normality using the Shapiro–Wilk test was used, demonstrating that the variables were normally distributed and allowing for parametric analysis of all recorded dependent variables. Furthermore, checking for covariance homogeneity using Levene's test indicated no significant difference with *p*-values > 0.05. As a result, a mixed-design MANOVA with a threshold of 0.05 was used to contrast the investigated variables at different-testing groups and measuring intervals. According to the power analysis, the sample size was calculated using G^*^power software (G^*^power 3.0.10). Analysis showed that 25 patients per group were sufficient to obtain a power level of 95% [power (1 error P) =0.95, =0.05, effect size =0.43]. This effect size was selected since it resulted in a manageable sample size (32). We assessed 115 individuals over the course of the 12-week research because we were concerned about losing some of them to follow-up (Figure 1).

## Results

A total of 115 patients were eligible for inclusion, and 100 of them were randomly selected for the study intervention (Figure 1). Group A (Diabetic foot ulcer group) included 25 patients who received conservative medical ulcer care and phonophoresis with topical BV, while group B (Diabetic Ulcer control group) included 25 patients who received conservative medical ulcer care and sham ultrasound sessions with only plain gel and no BV gel. Group C (Venous ulcer group) included 25 patients who received conservative medical ulcer care and phonophoresis with topical BV, while group D (Venous ulcer control group) included 25 patients who received conservative medical ulcer care and sham ultrasound sessions with only plain gel and no BV gel. As per participants' demographics in the three tested groups, there were no significant differences (*p* > 0.05) in the mean values of age between groups at the study baseline, showing homogeneity of the study sample (Table 1).

**Table 1:** Patients' demographics, including age, for the three tested groups at baseline.

	**Mean** ± **SD**^**a**^ **or frequency**					
	**Group A**	**Group B**	**Group C**	**Group D**	**Statistical values** ^b^	***P*** **-value** ^c^
Age	51.12 + 6	53.68 + 7.65	50 + 5.10	48.84 + 6.34	2.657	0.53
Time of injury	3.96 ± 0.889	4.2 ± 1.08	3.8 ± 0.816	3.92 ± 0.909	1.874	0.599
Diabetes	0	25	0	25	99	<0.0001
Obesity	7	9	12	10	2.185	0.535
Arterial disease	2	4	3	5	1.645	0.649
Atherosclerotic	2	4	4	6	2.357	0.502
High pressure	18	14	13	16	2.455	0.483
Venous surgery	1	2	6	1	7.48	0.058
Smoking	6	6	10	13	6.049	0.109

^a^SD, standard deviation.

^b^Statistical values are F-value for scale variables and the Kruskal–Wallis for non-parametric statistics.

^c^Significance level (0.05).

Based on the mean values of all outcome measures when comparing results before intervention (P_0_), after 6 weeks from the interventions (P_1_), and then after 12 weeks from the interventions (P_2_) of the intervention in group A, group B, and group C, there was a statistically significant reduction (*P* < 0.05) in the wound surface area (WSA). In group D, there were statistically significant lower WSA mean values in P_2_ vs. P_0_, while there was no statistically significant difference between P_1_ vs. P_0_ and P_1_ vs. P_2_. As regards the ulcer volume measurement (UVM; Tables 2, 3), the mean values of (P_1_) and (P_2_) in group A and group C showed statistically significant lower values than those of (P_0_), as well as lower mean values of (P_2_) than those of (P_1_). In group B, there was a statistically significant lower mean value of UVM at P_1_ and P_2_ than at P_0_, while there was no statistically significant difference between P_1_ and P_2_. In group D, there was no statistically significant difference between P_0_ vs. P_1_, P_1_ vs. P_2_, or P_2_ vs. P_0_. Regarding Ki67%, there were statistically significant higher mean values at P_2_ than at P_0_ in group A and group C, while there were no statistically significant differences between group B and group D.

**Table 2:** Mean and standard deviation for outcome measures in the three measuring periods in each tested group.

**Variables**	**Group**	**Mean** ± **SD**		
	**(*****n*** = **30)**	**P**_0_	**P**_1_	**P**_2_
Wound surface area (WSA) in cm^2^	Group A	6.65 + 1.25	4.65 + 1.25	2.56 + 1.28
	Group B	6.2 + 1.57	5.67 + 1.45	5.26 + 1.41
	Group C	6.58 ± 1.5	4.58 ± 1.92	2.39 ± 1.38
	Group D	6.54 ± 1.43	6.21 ± 1.38	5.87 ± 1.31
Ulcer volume measurement (UVM) in cm^3^	Group A	15.69 ± 3.27	11.61 ± 3.9	6.29 + 3.15
	Group B	15.34 ± 3.8	14.49 ± 3.39	13.85 ± 3.2
	Group C	10.8 ± 2.33	6.7 ± 2.94	2.96 ± 0.99
	Group D	12.55 ± 1.6	10.86 ± 1.61	10.33 ± 1.63
KI	Group A	3.09 ± 1.53		17.56 + 6.03
	Group B	3.38 ± 1.18		3.28 ± 1.17
	Group C	2.81 ± 1.48		22.72+7.07
	Group D	3.02 ± 1.03		3.13 ± 1.02

Group (A), Diabetic foot ulcer; Group (B), Control group; Group (C), Venous ulcer; Group (D), Control group.

P_0_, measurements at baseline; P_1_, measurements after 6 weeks from the interventions; P_2_, measurements after 12 weeks from the interventions.

**Table 3:** Multiple pairwise comparisons among the three measuring periods for primary outcomes at each study group.

**Variables**	**Group**	**P_0_-P_1_**		**P_0_-P_2_**		**P_1_-P_2_**	
		**MD**	**%**	**MD**	**%**	**MD**	**%**
Wound surface area (WSA) in cm^2^	Group A	1.91^**^	28.72	4^**^	60.15	2.09^**^	44.94
	Group B	0.53^**^	8.54	0.94^**^	15.16	0.407^**^	7.17
	Group C	2^**^	30.39	4.19^**^	63.67	2.19^**^	47.81
	Group D	0.33	5.04	0.67^*^	10.24	0.34	5.45
Ulcer volume measurement (UVM) in cm^3^	Group A	4.07^**^	25.94	9.4^**^	59.91	5.33^**^	45.90
	Group B	0.84^**^	5.47	1.49^*^	9.71	0.641	4.42
	Group C	4.11^**^	38.01	7.8^**^	72.63	3.7^**^	55.84
	Group D	1.69^**^	13.45	2.22^**^	17.66	0.53	4.86
KI	Group A		−14.46^**^	467	
	Group B		0.1	29.58	
	Group C		−19.91^**^	708	
	Group D		−0.11	3.64	

Group (A), Diabetic foot ulcer; Group (B), Control group; Group (C), Venous ulcer; Group (D), Control group.

MD, mean difference; %, percentage of improvement; P_0_, measurements at baseline; P_1_, measurements after 6 weeks from the interventions; P_2_, measurements after 12 weeks from the interventions.

^*^Significant difference (P ≤ 0.05).

^**^A highly significant difference (P ≤ 0.001).

A between-group comparison showed statistically significant lower mean values of the wound surface area (WSA) and ulcer volume measurement (UVM; *P* < 0.05) after 12 weeks of intervention in favor of group B and group D than in group A and group C, respectively (Tables 2, 4). WSA did not show a statistically significant difference in group A vs. group B after 6 weeks of treatment (P_1_), while it showed a statistically significant lower UVM in group A vs. group B at the same period. A comparison between group A and group C showed no statistically significant differences in WSA and UVM, while there were higher Ki67% mean values in group C after treatment.

**Table 4:** Multiple pairwise comparisons for outcomes between both tested groups.

**Variable**	**Groups in comparison**	**Assessment times**		
***P*-value**		**P_0_**	**P_1_**	**P_2_**
Wound surface area (WSA) in cm^2^	Group A vs. Group B	0.98	0.112	0.0001^**^
	Group C vs. Group D	0.99	0.002^*^	0.0001^**^
	Group A vs. Group C	0.998	0.998	0.99
Ulcer volume measurement (UVM) in cm^3^	Group A vs. Group B	0.98	0.008^**^	0.0001^**^
	Group C vs. Group D	0.209	0.0001^**^	0.0001^**^
	Group A vs. Group C	0.0001^**^	0.0001^**^	0.0001^**^
KI	Group A vs. Group B	0.469		0.0001^**^
	Group C vs. Group D	0.558		0.0001^**^
	Group A vs. Group C	0.998		0.001^**^

Group (A), Diabetic foot ulcer; Group (B), Control group for group A; Group (C), Venous ulcer; Group (D), Control group for group C.

P_0_, measurements at baseline; P_1_, measurements after 6 weeks from the interventions; P_2_, measurements after 12 weeks from the interventions.

^*^Significant difference across the measuring times (P ≤ 0.05).

^**^A highly significant difference across the measuring times (P ≤ 0.001).

## Discussion

In this trial, the therapeutic efficacy of BV phonophoresis was compared between groups A, B, C, and D in enhancing the healing of wounds caused by venous and diabetic foot ulcers. There was a substantial difference in all outcome markers within each group before and after treatment. The comparison of the WSA and UVM values between the four groups after 6 and 12 weeks of treatment revealed a highly substantial difference in both study groups (groups A and C) in comparison with their control groups (groups B and D), respectively.

This improvement could be attributed to the application of BV phonophoresis. Furthermore, in the case of hyperglycemia-induced skin lesions, treatment with BV phonophoresis may give considerable advantages.

The results of the current study agree with those of Amin and Abdel-Raheem (33) and Amin et al. (34), which revealed that, in diabetic rats, a wound dressing containing BV improved and sped up the healing of diabetic lesions. According to Han et al. (10), BV decreased TGF-1, fibronectin, and VEGF mRNA levels while increasing collagen type I mRNA levels. According to histological studies (35), type I collagen promoted wound healing in an animal model because the wound's diameter was minimized, and the repair capacity of BV was determined to be excellent.

The anti-inflammatory impact of 6% bee venom chitosan films was equivalent to that of indomethacin and hastened wound healing (36). Another study found that inflammation-induced cytokines were released in infected wounds and helped to decrease keratinocyte growth (12, 37). In diabetic rabbits, bee venom cross-linked to a hydrogel reduced inflammatory response and IL-6 generation while increasing collagen synthesis for wounds (34). This improvement could be due to bee venom containing antimicrobial characteristics (34, 35, 38). Another previous study showed antibacterial action against *Escherichia coli* and *Staphylococcus aureus* (39). Another research investigated the antioxidant properties of bee venom (40, 41). It reduced the degree of ROS-induced oxidative damage by inhibiting IL-1. A decrease in the inflammatory phase within the first 3 days after damage has been shown to be linked to poor wound healing (42). Another study found that BV is not cytotoxic at concentrations below 100 g/mL and that topical administration sped up cell renewal and wound healing (43). Bee venom has also been found to assist in the recruitment of bone marrow-derived endothelial cells and speed up wound healing in diabetic mice by decreasing oxidative stress mediated by activated transcription factor-3 (ATF-3) and inducible nitric oxide synthase (iNOS). Bee venom has also been shown to assist in the recruitment of bone marrow-derived endothelial cells, speeding up re-epithelialization and tissue remodeling (44). According to Hozzein et al. (45), bee venom raised the component of collagen type I and beta-defensin-2 in diabetic animal wounds, which plays a crucial function in wound closure regulation. It also stimulates angiogenesis by reactivating endothelial-specific receptor tyrosine kinase signaling, which plays an important role in wound healing regulation.

As a result, the utilization of bee venom, which has anti-inflammatory properties and enhances immune reaction, may aid in wound repair by augmenting the inflammatory stimulus essential for diabetic wound repair. Our laboratories are presently putting this notion to the test. Bee venom certainly tends to help wound repair due to its anti-inflammatory, antimicrobial, analgesic, and antioxidant characteristics. It may trigger the healing process by stimulating the inflammatory system and cytokines in chronic unhealed wounds.

In the current trial, there was no statistically significant difference in WSA and UVM between both study groups, A and C, after treatment. On the other hand, Ki-67 immunohistochemistry findings showed a higher proliferation rate after treatment in the venous ulcer study group than in the diabetic ulcer study group. This can be explained by the finding of Andriessen et al. (46) that chronic venous ulcers do not inhibit epidermal proliferation, and cytokeratin 16 is highly expressed in all of these ulcers. Furthermore, these *in vivo* studies showed that the recruitment of G0 cells into the cell cycle is not reduced in chronic venous ulcers, indicating that epidermal proliferation is not a limiting factor in the healing process.

Poor healing of venous ulcers may be a sign of a more severe underlying venous illness, but it is also possible that fibroblasts have aged and are less responsive to proliferative stimuli as a result of extended exposure to the environment of chronic venous ulcers (47–49). The average healing time for diabetic foot ulcers was 77.7 days (50), while the average healing time for venous ulcers varied: there was a 17% chance of the ulcer healing in 40 days, a 47% chance in 80 days, and a 77% chance in 120 days (51).

### Limitations of the study

Psychological and cultural concerns might restrict the study's conclusions. We could not achieve double-blinding or confirm if participants were following both their prescribed diet and correctly taking their medications to control blood glucose. More thorough studies determining the follow-up of different multiple therapeutic periods would be of extreme importance, in addition to diversity in the participants and their implications on the rate of recovery. Other kinds of ulcers should be studied in a similar manner. With improved data analysis and over a longer length of time than 3 months, more research should be done on a larger set of patients. A comparison of various physiotherapeutic modalities and protocols might be done in future studies. Further studies should be conducted using different parameters of ultrasound (intensity, frequency, and duration of treatment) or with other physical therapy modalities, such as iontophoresis instead of phonophoresis. Further studies are needed to compare the effectiveness of the treatment, after adjusting for factors related to sex and age and different concentrations of bee venom gel.

## Conclusion

In the treatment of chronic unhealed lower limb ulcers, BV administered by phonophoresis has a positive impact on the healing rate of venous and diabetic foot ulcers, as proven by the highly significant decrease in WSA and UVM and the highly significant increase in Ki-67 immunohistochemistry. We suggest adding this technique to the chronic ulcer treatment program and conducting follow-up studies for any further improvement.

## Data availability statement

The raw data supporting the conclusions of this article will be made available by the authors, without undue reservation.

## Ethics statement

The studies involving human participants were reviewed and approved by Research Ethical Committee, Faculty of Physical Therapy, Cairo University. The patients/participants provided their written informed consent to participate in this study.

## Author contributions

EO, HH, ZA, GM, GA, and AK: conception and design of the study. EO, HH, AA, and AK: writing original draft preparation and statistical analysis. ZA, GM, AA, and GA: writing reviews the discussion part. All authors contributed to manuscript revision, read, and approved the submitted version.

## References

[1] Tonnesen MG Feng X Clark RA. Angiogenesis in wound healing. J Investig Dermatol Symp Proc. (2000) 5:40–6. 10.1046/j.1087-0024.2000.00014.x 11147674

[2] Casey G. Causes and management of leg and foot ulcers. Nurs Standard. (2004) 18:57. 10.7748/ns2004.07.18.45.57.c3653 15305817

[3] González-Consuegra RV Verdú J. Quality of life in people with venous leg ulcers: An integrative review. J Adv Nurs. (2011) 67:926–44. 10.1111/j.1365-2648.2010.05568.x 21241355

[4] Morgan C Nigam Y. Naturally, derived factors and their role in the promotion of angiogenesis for the healing of chronic wounds. Angiogenesis. (2013) 16:493–502. 10.1007/s10456-013-9341-1 23417553

[5] Frykberg Robert G. Challenges in the treatment of chronic wounds. Adv Wound Care. (2015) 2015:635. 10.1089/wound.2015.0635 26339534 PMC4528992

[6] Moulik PK Mtonga R Gill GV. Amputation and mortality in new-onset diabetic foot ulcers stratified by etiology. Diabetes Care. (2003) 26:491–4. 10.2337/diacare.26.2.491 12547887

[7] Bajpai A Nadkarni S Neidrauer M Weingarten MS Lewin PA Spiller KL. Effects of non-thermal, non-cavitational ultrasound exposures on human diabetic ulcer healing and inflammatory gene expression in a pilot study. Ultrasound Med Biol. (2018) 44:2043–9. 10.1016/j.ultrasmedbio.2018.05.011 29941215 PMC6105501

[8] Eskridge EM Elliott WB Elliott AH Eskridge PB Doerr JC Schneller N. Adaptation of the electrical stimulation procedure for the collection of vespid venoms. Toxicon. (1981) 19:893–7. 10.1016/0041-0101(81)90087-8 7336452

[9] Kwon YB Lee HJ Han HJ Mar WC Kang SK Yoon OB. The water-soluble fraction of bee venom produces antinociceptive and anti-inflammatory effects on rheumatoid arthritis in rats. Life Sci. (2002) 71:191–204. 10.1016/S0024-3205(02)01617-X 12031688

[10] Han S Lee K Yeo J Kim W Park K. Biological effects of treatment of an animal skin wound with honeybee (*Apis melifera*. L) venom. J Plast Reconstr Aesthetic Surg. (2011) 64:e67–72. 10.1016/j.bjps.2010.08.022 20943448

[11] Heo Y Pyo MJ Bae SK Lee H Kwon YC Kim JH. Evaluation of phototoxic and skin sensitization potentials of PLA2-free bee venom. Evid Bas Complement Alternat Med. (2015) 2015:157367. 10.1155/2015/157367 26347784 PMC4546966

[12] Han SM Lee GG Park KK. Acute dermal toxicity study of bee venom (*Apis mellifera* L.) in rats. Toxicol Res. (2012) 28:99–102. 10.5487/TR.2012.28.2.099 24278595 PMC3834407

[13] SangMi H KwangGil L JooHong Y HaJu B Kwankyu P. Antibacterial and anti-inflammatory effects of honeybee (*Apis mellifera*) venom against acne-inducing bacteria. J Med Plants Res. (2010) 4:459–64. 10.5897/JMPR09.427

[14] Chen J Lariviere WR. The nociceptive and anti-nociceptive effects of bee venom injection and therapy: A double-edged sword. Progr Neurobiol. (2010) 92:151–83. 10.1016/j.pneurobio.2010.06.006 20558236 PMC2946189

[15] Ay S Dogan SK Evcik D Başer ÖÇ. Comparison the efficacy of phonophoresis and ultrasound therapy in myofascial pain syndrome. Rheumatol Int. (2011) 31:1203–8. 10.1007/s00296-010-1419-0 20354859

[16] Yang J Hu J He B Cheng Y. Transdermal delivery of therapeutic agents using dendrimers (US20140018435A1): A patent evaluation. Expert Opin Ther Pat. (2015) 25:1209–14. 10.1517/13543776.2015.1044974 26150049

[17] Zhao M Bai J Lu Y Du S Shang K Li P. Anti-arthritic effects of microneedling with bee venom gel. J Trad Chin Med Sci. (2016) 3:256–62. 10.1016/j.jtcms.2016.09.005

[18] Lai BL Wang LS Zhang S Tian Y. The advance in Chinese medicine gel. Tradit Chin Drug Res Clin Pharmacol. (2010) 21:211.

[19] Kloth LC. Electrical stimulation for wound healing: A review of evidence from in vitro studies, animal experiments, and clinical trials. Int J Low Extrem Wounds. (2005) 4:23–44. 10.1177/1534734605275733 15860450

[20] Gschwandtner ME Ehringer H. Microcirculation in chronic venous insufficiency. Vasc Med. (2001) 6:169–79. 10.1177/1358836X0100600308 11789972

[21] Olyaie M Rad FS Elahifar MA Garkaz A Mahsa G. High-frequency and noncontact low-frequency ultrasound therapy for venous leg ulcer treatment: A randomized, controlled study. Ostomy Wound Manage. (2013) 59:14–20. 23934374

[22] Ennis WJ Formann P Mozen N Massey J Conner-Kerr T Meneses P. Ultrasound therapy for recalcitrant diabetic foot ulcers: Results of a randomized, double-blind, controlled, multicenter study. Ostomy Wound Manag. (2005) 51:24. 16234574

[23] Samuels JA Weingarten MS Margolis DJ Zubkov L Sunny Y Bawiec CR. Low-frequency (<100 kHz), low-intensity (<100 mW/cm^2^) ultrasound to treat venous ulcers: A human study and *in vitro* experiments. J Acoust Soc Am. (2013) 134:1541–7. 10.1121/1.4812875 23927194 PMC3745491

[24] Kim SK Kim MC. The affect on delayed onset muscle soreness recovery for ultrasound with bee venom. J Phys Therapy Sci. (2014) 26:1419–21. 10.1589/jpts.26.1419 25276027 PMC4175248

[25] Cattoretti G Becker MH Key G Duchrow M Schlüuter C Galle J. Monoclonal antibodies against recombinant parts of the Ki-67 antigen (MIB 1 and MIB 3) detect proliferating cells in microwave-processed formalin-fixed paraffin sections. J Pathol. (1992) 168:357–63. 10.1002/path.1711680404 1484317

[26] Sato T Abe T Ichioka S. Factors impairing cell proliferation in the granulation tissue of pressure ulcers: Impact of bacterial burden. Wound Repair Regener. (2018) 26:284–92. 10.1111/wrr.12675 30265416

[27] Hunt KJ Valentine MD Sobotka AK Lichtenstein LM. Diagnosis of allergy to stinging insects by skin testing with Hymenoptera venoms. Ann Intern Med. (1976) 85:56–9. 10.7326/0003-4819-85-1-56 59564

[28] Nassify M. In-vitro and Therapeutic Effectiveness Evaluation of Some Preparations of Bee Venom. (MSc Thesis), Al-Azhar University, Cairo, Egypt (2014).

[29] Keltie K Reay CA Bousfield DR Cole H Ward B Oates CP. Characterization of the ultrasound beam produced by the MIST therapy, wound healing system. Ultrasound Med Biol. (2013) 39:1233–40. 10.1016/j.ultrasmedbio.2012.10.022 23562019

[30] Kim HW Kwon YB Ham TW Roh DH Yoon SY Kang SY. General pharmacological profiles of bee venom and its water soluble fractions in rodent models. J Vet Sci. (2004) 5:309–18. 10.4142/jvs.2004.5.4.309 15613814

[31] Elgohary HM Al Jaouni SK Selim SA. Effect of ultrasound-enhanced Nigella sativa seeds oil on wound healing: An animal model. J Taibah Univ Med Sci. (2018) 13:438–43. 10.1016/j.jtumed.2018.02.008 31435359 PMC6694951

[32] Welkowitz J Cohen BH Ewen RB. Introductory Statistics for the Behavioral Sciences. New York, NY: John Wiley & Sons (2006).

[33] Amin MA Abdel-Raheem IT. Accelerated wound healing and anti-inflammatory effects of physically cross linked polyvinyl alcohol–chitosan hydrogel containing honey bee venom in diabetic rats. Arch Pharm Res. (2014) 37:1016–31. 10.1007/s12272-013-0308-y 24293065

[34] Amin MA Abdel-Raheem IT Madkor HR. Wound healing and anti-inflammatory activities of bee venom-chitosan blend films. J Drug Deliv Sci Technol. (2008) 18:424–30. 10.1016/S1773-2247(08)50082-7

[35] Fennell JF Shipman WH Cole LJ. Antibacterial action of melittin, a polypeptide from bee venom. Proc Soc Exp Biol Med. (1968) 127:707–10. 10.3181/00379727-127-32779 4870538

[36] Gould L Abadir P Brem H Carter M Conner-Kerr T Davidson J. Chronic wound repair and healing in older adults: Current status and future research. Wound Repair Regener. (2015) 23:1–13. 10.1111/wrr.12245 25486905 PMC4414710

[37] Maas-Szabowski N Shimotoyodome A Fusenig NE. Keratinocyte growth regulation in fibroblast cocultures *via* a double paracrine mechanism. J Cell Sci. (1999) 112:1843–53. 10.1242/jcs.112.12.1843 10341204

[38] Fennell JF Shipman WH Cole LJ. Antibacterial Action of a Bee Venom Fraction (Melittin) Against a Penicillin-Resistant Staphylococcus and Other Microorganisms. San Francisco, CA: Naval Radiological Defense Lab. (1967). 10.21236/AD0658324 5300771

[39] Han S Yeo J Baek H Lin SM Meyer S Molan P. Postantibiotic effect of purified melittin from honeybee (*Apis mellifera*) venom against *Escherichia coli* and *Staphylococcus aureus*. *J Asian Nat Prod Res*. (2009) 11:796–804. 10.1080/10286020903164277 20183327

[40] Rekka E Kourounakis L Kourounakis P. Antioxidant activity of and interleukin production affected by honey bee venom. Arzneimittel-forschung. (1990) 40, 912–913. 2242083

[41] Suh SJ Kim KS Kim MJ Chang YC Lee SD Kim MS. Effects of bee venom on protease activities and free radical damages in synovial fluid from type II collagen-induced rheumatoid arthritis rats. Toxicol In Vitro. (2006) 20:1465–71. 10.1016/j.tiv.2006.06.016 16989977

[42] Badr G Badr BM Mahmoud MH Mohany M Rabah DM Garraud O. Treatment of diabetic mice with undenatured whey protein accelerates the wound healing process by enhancing the expression of MIP-1α, MIP-2, KC, CX3CL1 and TGF-β in wounded tissue. BMC Immunol. (2012) 13:1–9. 10.1186/1471-2172-13-32 22708778 PMC3676145

[43] Han SM Park KK Nicholls YM Macfarlane N Duncan G. Effects of honeybee (*Apis mellifera*) venom on keratinocyte migration *in vitro*. Pharmacogn Mag. (2013) 9:220. 10.4103/0973-1296.113271 23930005 PMC3732424

[44] Badr G Hozzein WN Badr BM Al Ghamdi A Saad Eldien HM Garraud O. Bee venom accelerates wound healing in diabetic mice by suppressing activating transcription factor-3 (ATF-3) and inducible nitric oxide synthase (iNOS)-mediated oxidative stress and recruiting bone marrow-derived endothelial progenitor cells. J Cell Physiol. (2016) 231:2159–71. 10.1002/jcp.25328 26825453

[45] Hozzein WN Badr G Badr BM Allam A Al Ghamdi A Al-Wadaan MA. Bee venom improves diabetic wound healing by protecting functional macrophages from apoptosis and enhancing Nrf2, Ang-1 and Tie-2 signaling. Mol Immunol. (2018) 103:322–35. 10.1016/j.molimm.2018.10.016 30366166

[46] Andriessen MP Van Bergen BH Spruijt KI Go IH Schalkwijk J Van De Kerkhof PC. Epidermal proliferation is not impaired in chronic venous ulcers. Acta Derm Venereol. (1995) 75:459–62. 8651025 10.2340/0001555575459462

[47] Lal BK Saito S Pappas PJ Padberg FT Jr Cerveira JJ Hobson II. Altered proliferative responses of dermal fibroblasts to TGF-β1 may contribute to chronic venous stasis ulcer. J Vasc Surg. (2003) 37:1285–93. 10.1016/S0741-5214(02)75295-6 12764277

[48] Mendez MV Raffetto JD Phillips T Menzoian JO Park HY. The proliferative capacity of neonatal skin fibroblasts is reduced after exposure to venous ulcer wound fluid: A potential mechanism for senescence in venous ulcers. J Vasc Surg. (1999) 30:734–43. 10.1016/S0741-5214(99)70113-8 10514213

[49] Seidman C Raffetto JD Marien B Kroon C Seah CC Menzoian JO. bFGF-induced alterations in cellular markers of senescence in growth-rescued fibroblasts from chronic venous ulcer and venous reflux patients. Ann Vasc Surg. (2003) 17:239–44. 10.1007/s10016-003-0004-3 12704538

[50] Zimny S Schatz H Pfohl M. Determinants and estimation of healing times in diabetic foot ulcers. J Diab Compl. (2002) 16:327–32. 10.1016/S1056-8727(01)00217-3 12200075

[51] Skene AI Smith JM Dore CJ Charlett A Lewis JD. Venous leg ulcers: A prognostic index to predict time to healing. Br Med J. (1992) 305:1119–21. 10.1136/bmj.305.6862.1119 1463945 PMC1883694

